# Protease-Activated Receptors in the Intestine: Focus on Inflammation and Cancer

**DOI:** 10.3389/fendo.2019.00717

**Published:** 2019-10-24

**Authors:** Morgane Sébert, Nuria Sola-Tapias, Emmanuel Mas, Frédérick Barreau, Audrey Ferrand

**Affiliations:** IRSD, INSERM (U1220), INRA, ENVT, UPS, Université de Toulouse, Toulouse, France

**Keywords:** protease-activated receptors (PARs), small intestine, colon, gut, inflammation, cancer

## Abstract

Protease-activated receptors (PARs) belong to the G protein-coupled receptor (GPCR) family. Compared to other GPCRs, the specificity of the four PARs is the lack of physiologically soluble ligands able to induce their activation. Indeed, PARs are physiologically activated after proteolytic cleavage of their N-terminal domain by proteases. The resulting N-terminal end becomes a tethered activation ligand that interact with the extracellular loop 2 domain and thus induce PAR signal. PARs expression is ubiquitous and these receptors have been largely described in chronic inflammatory diseases and cancer. In this review, after describing their discovery, structure, mechanisms of activation, we then focus on the roles of PARs in the intestine and the two main diseases affecting the organ, namely inflammatory bowel diseases and cancer.

## Introduction

Protease-activated receptors (PARs) belong to the family of G-protein coupled receptors (GPCRs). Their activation results from the specific cleavage, by proteases, of the amino terminal sequence that exposes a new N-terminal sequence as a tethered ligand, which then binds intramolecularly to activate the receptor. PARs are ubiquitous throughout the organism, although predominantly expressed in vascular, immune, intestinal epithelial cells and the nervous system. Thus, their activations regulate a set of crucial biological processes involved in physiology and diseases (1, 2).

In the intestine, cleavage and activation of PARs have been largely described in the modulation of pain (3), but are also linked to inflammation (3) and cancer (4–6). Indeed, the gastrointestinal tract being an important source of proteases, PARs might play crucial roles in multiple pathophysiological processes.

In polarized intestinal epithelial cells, these receptors are expressed at both apical and basolateral sides, suggesting that luminal, circulating and secreted proteases can reach and activate them (7). As a consequence, proteases coming from either coagulation cascade, inflammatory cells, microbiota, or intestinal epithelial cells are able to cleave and trigger PAR signaling to maintain gut homeostasis, regulate ion exchange, motility, permeability and healing mechanisms, but also lead to visceral hypersensitivity, inflammation, or cancer when upregulated (8–10).

## Protease-activated Receptors

### Discovery

Four PARs have been identified, PAR1, PAR2, PAR3, and PAR4, according to their cloning order (11). Surprisingly, the genes coding for PAR1, PAR2, and PAR3 are located on chromosome 5q, whereas PAR4 gene is located on chromosome 19p. These PARs are ubiquitous, with variable expression depending on the tissues and physiopathological context (12).

The first receptor of this family to be cloned was PAR1, in 1991. Originally, the authors wanted to identify the receptors involved in the mechanisms of action of thrombin in inflammation and haemostasis (13, 14). They ended discovering a new type of receptor activated, after proteolytic cleavage, by the protease.

The PARs cloning followed in 1994 with PAR2. After the isolation of a DNA sequence coding for a G-protein-coupled receptor from a mouse genomic library, the predicted protein displayed a structure similar to PAR1 and activated by a similar mechanism. This receptor was first described activated by trypsin, but not by thrombin. In addition, the authors described that an exogenous agonist peptide allowed the receptor activation, suggesting the importance of the proteolytic cleavage in this process. This receptor was indeed named PAR2 (15). Nowadays, it is established that thrombin can actually indirectly activate PAR2 by transactivation (16). More recently, in a murine PAR1 KO model, thrombin-induced PAR2 activation was described to trigger aortic vasodilatation and MAPK signaling (17).

A similar approach allowed PAR3 discovery in 1997. This thrombin-activated receptor seems to be a PAR4 cofactor (18). To date, no exogenous and specific agonist peptide has been shown able to induce its activation.

Finally PAR4 was discovered shortly afterwards. Both trypsin and thrombin can activate this receptor (19).

### Structure

**Figure 1:**
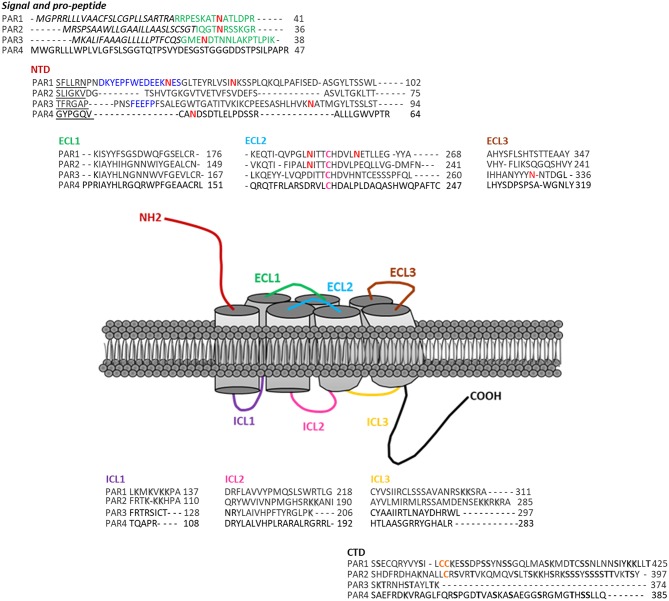
Protease-activated receptors structure. These receptors present several domains within their structures: the signal (italics lettering) and pro-peptide (green lettering) domains, a NH_2_-terminal domain (NTD), three extracellular loops (ECL1-3), three intracellular loops (ICL1-3), and a COOH-terminal domain (CTD). Within each receptor, the sequence of their specific tethered ligand are underlined. The blue lettering represents PAR1 and PAR3 Hirudin-like domains. Pink Cysteines are the ones forming a disulfide linkage between the transmembrane domain 3 and ECL2. PARs also present several post-translational modifications sites (N-glycosylation-red N, PAR1 and PAR2 putative palmitoylation sites–Orange lettering, Shadowed and bold lettering, respectively, represent ubiquitination and phosphorylation sites). Finally within PAR1 CTD, the YKKL motif is involved in the regulation of its trafficking.

The genes encoding the PARs consist in two exons. The exon 1 generates a larger N-terminal sequence than most GPCRs that includes the site of cleavage. The exon 2 codes for the receptor itself (20). PARs have seven transmembrane domains (TM), with an N-terminal domain of 17–26 amino acids, a pro-domain of 11–30 amino acids, 3 intracellular loops (ICL) and 3 extracellular loops (ECL), and a C-terminal domain of 13–51 amino acids. PAR1 and PAR3 display a “hirudin-like” domain, allowing a more specific binding for thrombin (13) (Figure 1). These receptors may also undergo post-transcriptional modifications (phosphorylation, ubiquitination, etc.). Each receptor can be stimulated by an activator ligand—TFLLR for PAR1, SLIGKV for PAR2, TFRGAP for PAR3, and GYPGQV for PAR4—followed by an amino acid sequence involved in the inhibition of its self-activation (21).

### Mechanisms of Activation

#### Canonical Activation

**Figure 2:**
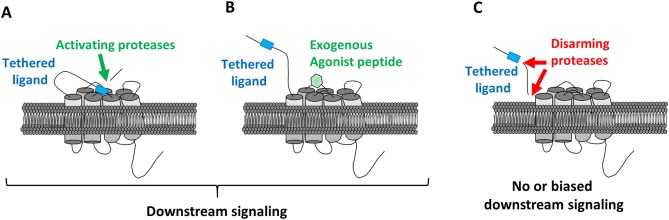
Mechanisms regulating protease-activated receptors activation. **(A)** Proteolytic cleavage by an activating proteases resulting in the binding of the tethered ligand to the ECL2, and downstream signaling activation. **(B)** Binding of an exogenous synthetic agonist peptide on ECL2 without proteolytic cleavage inducing downstream signaling activation. **(C)** No or Biased activation by disarming proteases cleaving the N-terminal domain after the tethered ligand either inhibiting the signal transduction or inducing biased downstream signaling compared to the one induced par the activating proteases. Moreover, disarmed receptors can be retained at the cell membrane making them available for a future activation by synthetic agonist peptides.

Under the action of a protease, the proteolytic cleavage of the receptor activator ligand within the canonical N-terminal domain site is irreversible (Figure 2A). This ligand will then bind to the second extracellular loop of the receptor, resulting in its conformational change that will induce signaling cascades (22).

PAR1 is cleaved (↓) at its canonical site, LDPR41 ↓ S42FLLRN, by thrombin (23).

PAR2 is activated at its canonical cleavage site, SKGR34 ↓ S35LIGKV, by trypsin (15).

PAR3 presents a putative cleavage site for thrombin, LPIK38 ↓ T39FRGAP (18). However, no signal transduction seems to result from this putative cleavage, and compared to other receptors, no protease able to activate PAR3 has been yet identified.

PAR4 can be cleaved by thrombin and trypsin, at similar doses, at this canonical site: PAPR47 ↓ G48YPGQV (19). PAR4 can also be activated by cathepsin G, plasmin, factor X and kallikreins (24–27).

Table 1 gives examples of activating proteases as well as synthetic peptides and tethered ligand sequences, but also the sites of expression and the induced effects for each of the PARs.

**Table 1:** PARs expression sites, activating proteases, tethered peptides, and main effects in the intestine.

	**PAR1**	**PAR2**	**PAR3**	**PAR4**
Sites of expression in the gut	Entero- and colonocytes, intestinal epithelial primitive cells, myenteric and submucosal neurons, fibroblats, smooth muscles, mast cells, immunes cells, endothelium, human colon epithelial cancer cells	Entero- and colonocytes, intestinal epithelial stem/progenitor cells, myenteric and submucosal neurons, fibroblats, smooth muscles, mast cells, immunes cells, endothelium, human colon epithelial cancer cells	Detected in non-identified cells in the small intestine	Entero- and colonocytes, enteric neurons, immune cells, endothelium, submucosa
Activating proteases	Thrombin, Factor VIIa, Factor Xa, Trypsin, MMP-1, MMP-2, MMP-3, MMP-8, MMP-9, MMP-12, MMP-13, MMP-14, Neutrophil elastase, Proteinase-3, Plasmin, Kallikrein-4,-5,-6, Kallikrein-14, Granzyme A, B, K, Calpain-1, Gingipain, cathepsin G	Trypsin, trypsin-2, trypsin-3, trypsin VI, mast cell tryptase, tissue factor, matriptase/membrane-type serine protease I, Factor Xa, Factor VIIa, gingipain, acrosin, elastase, Thrombin, Tryptase, Cathepsin G, Cathepsin S, Neutrophil elastase, Proteinase-3, Plasmin, Testisin, Kallikrein-4, Kallikrein-5,-6,-14, Calpain-2	Thrombin, trypsin, Factor Xa	Thrombin, trypsin, cathepsin G, Trypsin VI, Factor Xa, Factor VIIa, gingipain, Kallikrein 14
Tethered peptide sequences (human)	SFLLRN	SLIGKV	TFRGAP	GYPGQV
Effects in the gut	Apoptosis, cell proliferation, motility, increased permeability, ion secretion, smooth muscle contraction and relaxation, inflammation, prostaglandin release	Apoptosis, cell proliferation, motility, increased permeability, ion secretion, ion channel activation, smooth muscle contraction and relaxation, inflammation, prostaglandin and eicosanoid release, neuropeptide release, amilase secretion, neuronal hyperexcitability, visceral hypersensitivity, motor functions		Motor functions, colon cancer cell proliferation

#### Non-canonical or Biased Activation

Then, the notion of biased activation, characterized by a stimulation of the receptor on different sites than the canonical cleavage site, called “non-canonical sites,” appeared. This biased activation causes incomplete or different signaling compared to the ones observed after canonical activation. Proteases can activate PARs in a biased way (Figure 2C).

Biased activation was first described for PAR1 signaling (28). In this study, the authors demonstrated that MMP1 activates PAR1 *via* an activator ligand located two amino acids upstream of the one generated by thrombin (PRSFLLR ligand), but resulting in the same signaling as the one induced by thrombin. Another team showed a biased PAR1 activation leading to a different signaling. Indeed, PAR1 activation by activated protein C via a non-canonical site favors opposite effects than thrombin, i.e., anti-inflammatory effect and endothelial barrier protection (29). PAR1 can also be activated in a biased manner by other proteases and coagulation cascade actors, such as plasmin, factor X, granzymes A, trypsins, kallikreins, and cathepsin G (13, 25, 30–35).

Regarding PAR2, a study demonstrates the role of neutrophil elastase in MAPK signaling through biased activation of PAR2 (36). PAR2 can also be activated by other serine proteases, such as tryptase, granzymes, and kallikreins (23, 27, 37). To date, no studies demonstrating the biased activation of PAR3 and PAR4 have been reported.

#### Activation by Agonist Peptides

Thus, considering the diversity of elements able to cleave and activate the PARs, it has not been easy to decipher for each individual receptor its own mechanisms of activation. For example, thrombin can activate PAR1, PAR3, and PAR4. Deciphering the specific signaling triggered by PAR1 via thrombin is in consequence difficult. In that context, using synthetic peptide sequences or agonist peptides of 5–6 amino acids is paramount (12, 13, 38) (Figure 2B).

Several peptide sequences, with a different number of amino acids, additional hydrophilic residues or amino acid substitutions relative to the PAR1 activator ligand sequence, have been developed to activate PAR1. The most efficient one is in fact similar to PAR1 activator ligand sequence, TFLLR (39). Another point is the signaling induced by the agonist peptides. Indeed, it has been observed that the signaling generated via an agonist peptide is not identical in all respects to the one induced by proteolytic cleavage, confirming the biased activation. For example, several agonist peptides for PAR1 have shown various effects on signaling triggering platelet activation: no activation, little activation or complete activation (40). In addition, the MAPK pathway generated by the activation of PAR1 via thrombin is not triggered by the SFLLRN-NH_2_ agonist peptide (41) unless the doses of agonist peptides used are significantly higher (100-fold) than the commonly used doses (42).

Regarding PAR2, here again, depending on the peptide tested, the results are not identical. Indeed, PAR2 activation via the SLAAAA agonist peptide results in intracellular calcium release, MAPK pathway signaling and receptor internalization (43), whereas the SLAAAA-NH_2_ agonist peptide only induces intracellular calcium release (44). An activator sequence, SLIGKV, resulting in intracellular calcium release in rat and human cell lines was then validated (45–47). Next, further studies have allowed to design a more potent PAR2 agonist peptide by adding a seventh or eighth amino acid, leucine type (48). However, although these agonist peptides are stable, they display low bioavailability and low solubility.

No PAR3 specific agonist peptides have been generated. Indeed, the peptides designed with that aim, such as TFRGAP-NH_2_, seem actually to activate PAR4. An explanation could be a PAR3 and PAR4 dimerization as described in response to thrombin (49, 50).

Regarding PAR4, the agonist peptide GYPGQV-NH_2_ specifically activates the receptor, causing contractility of the aorta and longitudinal gastric muscles in the rat (51).

#### Disarming

PARs activation can be inhibited by disarming the receptor. Indeed, some proteases can prevent the canonical proteolytic cleavage by a proteolytic cleavage upstream of the activator ligand sequence of the receptor (Figure 2C). A second mechanism involves proteolytic cleavage within the receptor sequence to prevent signaling induction (52–54). For example, kallikrein 14 (KLK14), trypsin, cathepsin G, elastase, and plasmin disarm PAR1 (27, 31, 52, 55, 56). The disruption of PAR2 can be achieved by plasmin, PR3, elastase, and cathepsin G (57, 58).

#### Co-activation of PARs

**Figure 3:**
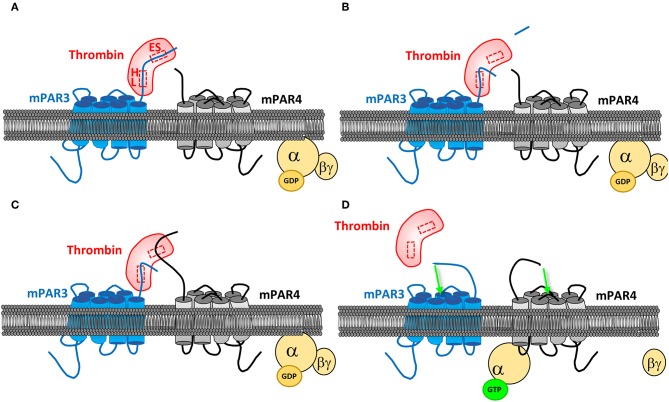
PAR4 co-activation by PAR3. **(A)** PAR3 binds thrombin active site via its cleavage site. PAR3 hirudin-like domain (HL) allows a more specific binding to the protease, on its exosite I (ES). **(B)** After PAR3 proteolytic cleavage by thrombin, which releases PAR3 N-terminal peptide, the receptor remains linked to the protease via the HL domain. **(C)** The active site of thrombin being free, it can bind to PAR4. **(D)** The PAR3:PAR4 heterodimerization results in a conformational change of both receptors and their activation, allowing them to couple to the G proteins and transduce signaling.

PARs can also be activated through co-activation or transactivation. Indeed, the hirudin-like domain present on the PAR1 and PAR3 sequences allows increasing the affinity of these receptors for thrombin, helping in turns to activate PAR4, which does not have such a domain (Figure 3). One study reports that the only expression of PAR3 in COS7 cells does not induce any signaling in response to thrombin, while co-transfecting PAR4, allowed an inositol triphosphate signaling as if they only expressed PAR4, but at a lower dose of thrombin. PAR4 co-activation with PAR3 was then confirmed (49). PAR3 binds thrombin through its exosite I, allowing the active site of thrombin to remain free and to activate other PARs. PAR3 then changes the conformation of thrombin and increases its affinity for PAR4. This mechanism has been described by crystallography (59). Activation of PAR4 via PAR1 has also been confirmed. By FRET, a study evidenced the heterodimerization of PAR1 and PAR4 in response to thrombin, this heterodimerization leading to an increased platelet aggregation compared to the one induced by PAR4 alone (60).

#### Transactivation

**Figure 4:**
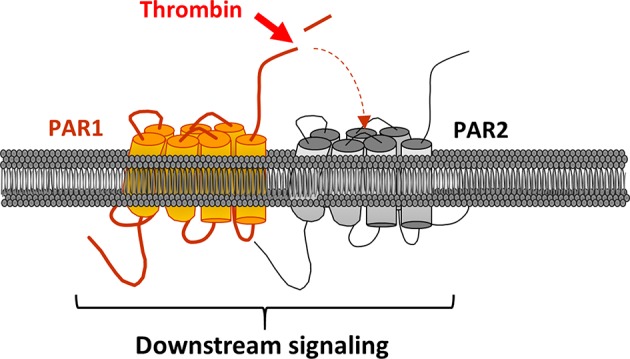
PAR2 transactivation via PAR1. PAR1 is clived and activated by thrombin. In turns, its activating ligand links PAR2 ECL2 leading to the receptor and downstream signaling activation.

PAR1 can also be activated by a mechanism called transactivation (Figure 4). The first study to highlight this phenomenon dates back to 1996 (61). Blackhart et al. wanted to characterize the specificity of PAR1 and PAR2 ligands (SFLLRN and SLIGKV) by analyzing the cross-reactivity of these receptors. In view of their sequence similarities, the authors looked at whether the PAR2 agonist peptide could activate PAR1. The results confirmed their hypothesis. The second step was to see whether thrombin could activate PAR2 through PAR1, as it is the case with the PAR1 agonist peptide. In this aim, researchers have mutated PAR1 making sure that it is still able to bind to thrombin via the exosite I, but without the thrombin being able to cleave PAR1 and cause signaling. The mutated PAR1 was then co-expressed with PAR2 in human endothelial cells. This coexpression caused signaling, similar to that caused by the individual expression of PAR1 and PAR2 in endothelial cells, after stimulation with thrombin (16). In order to confirm the activation of PAR2 via PAR1, a PAR1 antagonist was used, resulting in the inhibition of 75% of PAR1-induced signaling. The fact that the signal could be completely blocked with the addition of a PAR2 antagonist further supported this mechanism of activation between the two receptors.

In addition, this transactivation leads to ERK1/2 signaling (Extracellular signal-Regulated Kinases 1/2) and appears to be more prevalent during pathological events, such as sepsis in endothelial cells, chronic inflammation and carcinogenesis (62–64). FRET and co-immunoprecipitation approaches allowed to confirmed this mechanism of transactivation by heterodimerization (63).

## Proteases Activating PARs

Proteases, also known as proteinases and peptidases, are degradative enzymes for protein catabolism that hydrolyse a peptide bond to generate amino acids (65). The genes coding for proteases represent 2% of the mammalian genome and can be distributed in five groups depending on their mechanism of hydrolytic cleavage: serine-, metallo-, cysteine-, aspartate-, and threonine proteases. Serine-, Metallo-, and Cysteine-proteases are able to cleave and activate PARs, triggering different signaling pathways, although PAR4 is mainly activated by thrombin, while PAR2 is cleaved and activated by trypsin.

### Matrix Metalloproteinases (MMPs)

MMPs are a group of zinc- dependent endopeptidases known to degrade and remodel the components of the extracellular matrix. Depending on their substrate specificities, MMPs are subdivided into six groups: Collagenases, gelatinases, stromelysins, matrilysins, membrane-types MMPs, and non-classified MMPs. Besides the extracellular matrix turn over, MMPs are involved in other tissue maintenance functions, such as wound healing, and regulation of a broad range of molecules, such as chemokines, cytokines, growth factors, cytoskeleton, and junctional proteins (66). Dysregulation of MMP activity leads to the development of several pathologies including chronic inflammatory diseases as inflammatory Bowel Diseases (IBD) and cancer (67).

### Serine Proteases

Theses proteases are enzymes that hydrolyze peptide bonds within the protein sequence, in which serine serves as nucleophilic amino acid at the active site. Serine proteases, the most abundant group of proteases, are widely distributed in nature and present in the three domains of life (archaea, bacteria, and eukaryotes) as well as in viral genomes (68). Widespread throughout the human body, serine protease are usually endopeptidases that hydrolyse the peptide bond in the middle of a polypeptide chain. However, some are exopeptidases cleaving only terminal amino acid residue. Mammalian serine proteases comprise matriptase, plasminogen activators, chymotrypsin, trypsin, and proteolytic enzymes produced by polymorphonuclear cells, such as cathepsin G and neutrophil elastase. Bacteria from commensal microbiota are also an important source of proteases present in the GI tract.

### Cysteine Proteases

Most are found intracellularly. Besides their fundamental functions of catabolism and protein processing, cysteine proteases mediate other signaling pathways involved in programmed cell death, inflammation and intestinal mucosa integrity (epithelium turnover and homeostasis) (69, 70). Cysteine group comprises caspases, autophagins, calpains, and deubiquitinases intracellularly, and cathepsins B, K, and L extracellularly (9) Altered activity of cysteine proteases is associated with IBD (71).

## PARs and Intestinal Homeostasis

As mentioned above, the colon is highly exposed to proteases, whether pancreatic, bacterial proteases from resident colon cells or proteases produced and secreted by epithelial cells (72). These proteases intervene in a multitude of physiological and pathological processes, through activation of PARs (72). Although the roles of proteases in the colonic epithelium are not fully understood, PARs have been extensively studied with agonist peptides, even if these peptides do not always reflect in a precise way the functions of the PARs in the physiology and the pathology.

In the colon, smooth muscle cells, endothelial cells, enteric neurons, fibroblasts and immune cells, such as neutrophils, lymphocytes, macrophages, express PAR1. Originally, PAR1 expression was only reported in cancerous colon epithelial cells, but not in normal epithelial cells (4). An explanation is that, at that time, no anti-PAR1 antibody was effective. Since then, our team has described its, and PAR2, expression in normal human and murine colonic epithelium by immunostaining (73). The expression of PAR3 is poorly studied and has never been reported in the colon. PAR4 is expressed in colonic epithelial cells, at the submucosa level (10).

### Intestinal Secretions and PARs

One study demonstrated that PAR1 activation on intact cultured monolayers of intestinal epithelial cells in Using chambers resulted in chloride ion release, whereas in intact tissues, PAR1 activation would not result in this release (74). This difference between cultured cell monolayer and tissue would emphasize the importance of the location of the receptor on the cell. Indeed, it is thought that the activation of PAR1 on the apical side (toward the intestinal lumen), would have a protective role, generating a release of chloride ions, allowing a faster transit and the elimination of pathogens or at least of their toxins. Activation of PAR2 also promotes the secretion of chloride ions and is correlated with diarrhea in inflammatory conditions, as found in the tissues of IBD patients (75). In addition, other elements, such as amylase and mucin, are secreted in response to PAR2 activation (52).

### Intestinal Motility and PARs

Another colon function is to allow the transit of chime. This involves a regulation of the intestinal motility. The role of PAR1 and PAR2 in this process was demonstrated by the stimulation of circular and longitudinal rat colonic muscle layers with either thrombin, trypsin or agonist peptides. This study showed that both PAR1 and PAR2 activations result in muscle contraction and relaxation (76). The same authors demonstrated that the nervous system is involved in this regulation by secreting tachykinins (77).

### Sensory Function

PAR1 and PAR2 play a role in nociception. Thus, several studies have reported that colorectal distension performed by intracolonic administration of trypsin and PAR2 agonist peptide in rats caused visceral pain (78). PAR2 would have a pro-nociceptive role. Regarding PAR1, its role seems to be anti-nociceptive. Indeed, a study showed that the injection of PAR1 agonist into the mouse paw does not induce a response to a mechanical or thermal stimulus, but still increases the sensitivity threshold of the perception of pain (79). In addition, PAR1 would inhibit the transmission of nociceptive signals (80). Intracolonic administration of PAR4 agonist peptide decreased visceral motility, after colorectal distention and decreased pain induced by PAR2 activation (81).

### Intestinal Permeability

Activation of PARs present in epithelial cells leads to changes in paracellular permeability. Activation of PAR1, in response an agonist peptide, increases the intestinal permeability via the apoptotic process through caspase 3, and an alteration of ZO1 expression (82). Moreover, neutrophil proteases, such as elastase and PR3, via the basolateral activation of PAR1 and PAR2, can also modulate the intestinal permeability (83). Cenac et al. have shown that in mouse model, PAR2 activation by trypsin, tryptase, and chymase (all from serine proteases family) promotes an increase in colonic permeability displaying inflammation and disruption on the intestinal barrier integrity (84). These results have been supported by other studies, which have shown an alteration on the intestinal permeability using PAR2 agonists (78, 85). The mechanism of action through PAR2-mediated modification of intestinal permeability involved the calmodulin and MLCK. PAR2 agonist, via the calmodulin, increases MLCK phosphorylation, which leads to epithelial cell cytoskeleton contraction and the enhancement of the mucosal permeability. ML-7, an MLCK inhibitor, abolished the disruption of the tight junctions composition and function (86). Another study revealed that activation of ERK1/2 by tryptase, in cultured colonocytes, also phosphorylates MLCK, leading to epithelial cells disruption (87). Trypsin-3, released by intestinal epithelial cells in response to LPS, is able to cleave and activate PAR2 to increase the intestinal permeability (88). Thus, these results also highlight the involvement of PARs in intestinal inflammation.

### Survival and Proliferation

PAR1 and PAR2 have been both described involved in the stimulation of colorectal cancer cell proliferation (4, 89). In addition, activation of PAR2 leads to reduced apoptosis of colonic epithelial cells by activating MEK1/2 and PI3K (90). PAR2 activation also regulates the survival of colonic stem cells in the organoid model via the GSK3β pathway (73). More recently, in human colon organoid cultures, we reported that thrombin significantly reduces the size of budding structures, metabolic activity and proliferation, while increasing apoptosis. In the same study, we reported that both PAR1 and PAR4 antagonists inhibited apoptosis regardless of thrombin doses (91).

## PARs in Inflammatory Bowel Diseases

### Inflammatory Bowel Diseases (IBD)

The IBD, Crohn's disease (CD) and ulcerative colitis (UC), are chronic diseases causing inflammation of the gut (92). Since the second part of the last century, IBD has emerged as a public health challenge worldwide. In North America and Europe, more than 1.5 million people exhibit these pathologies, respectively (93). Outside the industrialized countries, the number of people affected by IBD remains unclear. Recently, the highest reported prevalence values for UC and CD were reported in Europe and North America (94). Although the molecular mechanisms of IBD are poorly understood, recent data suggest that IBD occurs in genetically predisposed individuals developing an abnormal immune response to intestinal microbes after being exposed to specific environmental triggers (95). Moreover, stools from IBD patients showed increased levels of active proteases, which are secreted meanly by infiltrated and resident cells, intestinal epithelial cells or smooth muscle (7). In addition, increased fecal proteases in IBD might result from both commensal and pathogenic gut bacteria, which can secrete serine proteases, cysteine proteases and MMPs (96–98).

### PARs in IBD

In the gut, PARs are stimulated by endogenous proteases, such as pancreas trypsin, cells of the intestinal mucosa (immune cells including mast cells, epithelial cells including goblet, neuroendocrine, and enterocyte cells), or gut microbiota. Moreover, PARs expressions in the intestinal epithelium are different between IBD patients and healthy individuals. Colonic biopsies from UC and CD patients exhibited increased expression PAR1, while PAR2 and PAR4 are just upregulated in UC conditions. Enhanced levels of these receptors is linked to its activation-internalization-degradation signaling induced by proteases released from eukaryotic host cells but also from gut micro-organisms. Indeed, on neutrophil cells, *Candida albicans* induces a TLR2-dependent PAR1 stimulation and expression, while *Aspergillus fumigatus* inhibits a TLR4-dependent PAR2 activation and expression (99).

#### PAR1

PAR1 stimulation triggers apoptosis of the epithelial cells within the gut mucosa through a mechanism involving caspase-3 activation. This excessive apoptosis is associated to a disruption of the intestinal barrier function, promoting then the development and/or severity of the colitis (82). PAR1 is expressed by intestinal epithelial cells but also by endothelial cells, enteric neurons, myocytes, and immune cells (52). PAR1 expression by intestinal epithelium is linked to the presence of microbiota (100), and its colonic stimulation leads to colitis (10, 101). Moreover, deletion or blockage of PAR1 reduce inflammatory signs and mortality in a murine model of IBD (102). In addition to an increased PAR1 expression in IBD patient colons, it has been recently shown that thrombin level is increased in CD colonic biopsies (103). Moreover, an increased incidence of thrombosis has been observed in IBD patients (104). Altogether, these studies evidence a potential role for PAR1 in IBD pathophysiology, however it is still not clear whether the intestinal bacteria directly activate PAR1 through the release of proteases. Nevertheless, this notion has been evidenced by a study showing that a cysteine protease released by *Porphyromonas gingivalis* increases the expression of pro-inflammatory cytokines through PAR1 activation (105).

#### PAR2

PAR2 receptor is localized at the apical and basolateral membranes (84, 106, 107) of the gut epithelium cells and can be stimulated by trypsin, tryptase, and bacterial proteases (108). PAR2 is also present in the cells membrane of numerous immune cells, stromal cells or endothelial cells. Thus, PAR2-associated inflammation coming from numerous pathways from either systemic or local locations. Systemically, PAR2 stimulation promotes the rolling, adhesion and extravasation of leukocytes (109). Locally at colonic level, activation of this receptor triggers colitis (7). Additionally, blockage of PAR2 activation reduces the severity of the colitis induced by either TNBS or a PAR2 agonist (110). All together, the majority of the studies show that stimulation of PAR2 triggers an inflammatory response. Nevertheless, one study has evidenced a protective effect of chronic stimulation of PAR2 in a model of colitis (111). This protective effect might be the result of a local desensitization, or anti-inflammatory effects on macrophages (112). Additionally, although we do not know which of the numerous mechanisms described for PAR2 stimulation in the intestine triggers colitis in rodent models, excessive PAR2 stimulation by trypsin and tryptase has been speculated to mediate colitis in IBD patients. Moreover, in IBD, PAR2 expression is increased at the membrane of mast cells, then participating in PAR2-induced colitis (113). Thus, although, these studies have highlighted the major role played by PAR-2 in the colitis mediated by mast cells, recent papers have also reported that proteases coming from gut micro-organisms could participate to PAR2 activation altering then colonic homeostasis (96). PAR2 can be also stimulated by gut micro-organisms either directly by bacterial proteases, as demonstrated for *P. gingivalis* (108) or *C. difficile* (114), or indirectly by the release of host cells proteases triggered by bacteria stimulation (37). Moreover, as antibiotic treatment diminished intestinal PAR2 expression, this suggests that, in addition to its activation, PAR2 expression can also be regulated by the gut microbiota (115).

Altogether, these studies report the role for PAR2 in IBD pathophysiology. However, the most important source of the proteases activating PAR2 to promote IBD is largely unclear.

#### PAR3

The biological importance of PAR3 is not fully demonstrated. Thus, PAR3 does not exhibit, as the other PARs, a C-terminal intracytoplasmic tail. However, as described previously, PAR3 could play a role as co-factor or co-receptor for PARs and/or other receptors. Although PAR3 mRNA has been evidenced in the gut, no study has reported its involvement in intestinal inflammation (18).

#### PAR4

PAR4 expression has been detected in the gut (19) and on colonocytes (116). It can be cleaved and then activated by several proteases including thrombin, trypsin and by the neutrophil granule protease cathepsin G (24). PAR4 stimulation on leukocyte has been reported to promote their rolling and adherence, then suggesting a pro-inflammatory role (79). Moreover, colonic exposure to PAR4 agonists increases the paracellular permeability of the colic epithelium, suggesting that PAR4 could favor the genesis of IBD (117). In human colon, PAR4 expression is very weak in non-IBD patients, while its expression is drastically increased in UC patients. Interestingly, cathepsin G activity is enhanced in fecal supernatant from UC individuals compared to controls (117). Moreover, inhibition of this cathepsin G activity resulted in a restored gut paracellular permeability (117). Thus, cathepsin G, via PAR4 receptor, could participate in the increase of the gut permeability in UC patients.

## PARs in Colorectal Cancer

### Colorectal Cancer (CRC)

Worldwide, colorectal cancer is the second and the third most commonly occurring cancer in women and men, respectively. There were over 1.8 million new cases detected in 2018 (GLOBOCAN database 2018, http://gco.iarc.fr/). CRC is the fourth most common cause of death from cancer in the industrialized world. The survival depends on the stage of the pathology at the time of the diagnosis. Indeed, patients with the later-stage diagnosis have the poorer survival. Indeed, the 5-years survival rate is 90% for colorectal cancers diagnosed at an early stage but drops down to 13% for those diagnosed at a late stage.

Since a long time now, proteases have been associated with tumor progression mainly due to their ability to degrade the extracellular matrix (ECM), favoring thus tumor cell invasion and metastasis process (118). However, it is now well-established that their roles in cancer is not only restricted to their ECM degradation capacities, these enzymes acting directly on the cancer cells via the PARs.

### PARs in CRC

#### PAR1

Evidence that thrombin potentiated the tumor growth and the metastatic process *in vivo* was obtained in 1991. Nierodzik et al. treated cancer cell lines, including the murine carcinoma cell line CT26, with thrombin (0.5–1 U/mL). They then injected these cells intravenously in the mouse and observed an increase in the metastatic power of these cells (119). Nevertheless, a study shows that thrombin concentration may have opposite effects on tumor cells (120). Indeed, at a low dose of thrombin, 0.1–0.5 U/mL, PAR1 activation resulted in the growth of tumor cells. Whereas, at a higher dose of thrombin, 0.5–1 U/ml, growth was decreased in favor of cell apoptosis. Since, it has been demonstrated that PAR1 plays a direct role in the progression of epithelial colon tumors, regulating both cell proliferation and migration (4, 121, 122) have pro- and anti-apoptotic effects, depending on the dose of thrombin or agonist peptides used (123).

Patients with IBD are 10–20 times more likely to develop CRC (124). One study revealed the role of thrombin in the development of colon cancer from an inflammatory context. Using the murine DSS-induced colitis model and the colitis-associated cancer model treating the mice with DSS and AOM, the authors observed the formation of adenomas. The result obtained was a uniform development of adenomas from aberrant crypts in control mice (Factor II^+/+^ mice) and, in Factor II ^+/−^ mice, a decrease in these precancerous lesions as well as the number of adenomas (125). The involvement of thrombin in cancer development from a previous inflammatory state has been proven by treating the mice with hirudin, an inhibitor of thrombin, these mice displaying then a slower adenoma development. However, a recent study reports that PAR1-deficient APC^Min/+^ mice display an increased number of adenomas and larger adenomas than PAR1-expressing mice suggesting a protecting role of PAR1 in some CRC favoring context (126). Actually, the same authors already described earlier that the growth of colonic adenocarcinoma in PAR-1-deficient mice was decreased compared to control animals, and proposed an implication of the stromal cell-associated PAR-1 as target important for tumor development (127).

#### PAR2

Regarding the role of PAR2, several human colon cancer cell lines, namely T84, Caco-2, HT-29, and C1.19A, produce and secrete trypsin at concentrations compatible with PAR2 activation, supporting the idea of a possible autocrine/paracrine regulation of PAR2 activity by trypsin in colon cancer cells (128). Trypsin is produced by human cancer colon cells and activates protease-activated receptor-2 within these cells, depicting an autocrine loop. Interestingly, EGF-R transactivation by PAR2 results in the growth of colon cancer cells after the activation of a Src/ERK1/2 pathway (85).

More recently, we reported in three-dimensional cultures of murine colorectal crypt and in Caco-2 cells, that PAR2 activation decreases the numbers and the size of normal or cancerous spheroids. Spheroids deficient for display an increased proliferation, suggesting that cell proliferation is repressed by PAR2. However, in the same study, PAR2-stimulated normal cells are more resistant to stress, suggesting PAR2 pro-survival roles. Indeed, PAR2-deficient normal spheroids display an increase of active caspase-3. Moreover, we showed that PAR2, but not PAR1, was able to trigger GSK3β activation in normal and tumor cells. The PAR2-triggered GSK3β activation involves an arrestin/PP2A/GSK3β complex that is dependent on the activity of the Rho kinase. Finally, the survival of PAR2-stimulated cultures can be pharmacologically inhibited using a GSK3 inhibitor. This study highlighted the PAR2/GSK3β pathway as a novel critical player in the regulation of stem/progenitor cell survival and proliferation in normal colon crypts and colon cancer (73).

#### PAR3

To date, no clear role of PAR3 in CRC has been reported.

#### PAR4

Regarding PAR4, its expression is increased in colorectal cancer tissues compared to the associated normal tissues. This overexpression seems to promote colorectal cancer cell proliferation, survival and metastasis, making PAR4 a potential therapeutic target in CRC (129).

PAR4 mediates thrombin effects on human colon cancer cells. AP4, a specific PAR4 agonist, mimics the effects of thrombin on cell proliferation. Its effects on calcium mobilization in CHO-PAR4-expressing cells are similar to the one observed in HT-29 cells, while HT-29 cells treatment with a reverse peptide has no effect on calcium mobilization. Finally, AP4 promotes colon cancer cell proliferation. More recently, PAR4 overexpression in LoVo cells has been shown, via activation of the ERK1/2 pathway, to increase their proliferation and migration and tumorigenesis capacities, while its knock-down in HT29 results in opposite effect (129). Thus, PAR4 should be regarded as a crucial receptor by which thrombin modulates colon carcinogenesis (130).

## Conclusion and Futures Perspectives

Since their discovery in the early nineties, PARs have been shown to be largely involved in the regulation of the intestine physiological processes, but also in the two main diseases affecting the organ, namely inflammatory bowel diseases and colorectal cancer. Consequently, these G-protein coupled receptors represent attractive targets for therapeutic drug development. More than aiming to target their ligands, efforts to develop specific receptor inhibitors are currently regarded as a priority although to date, the development of effective PAR antagonist yet remains in its early stages.

## Author Contributions

MS, NS-T, FB, and AF wrote the manuscript. EM did a careful reading and review of the manuscript before its submission.

### Conflict of Interest

The authors declare that the research was conducted in the absence of any commercial or financial relationships that could be construed as a potential conflict of interest.
